# Role of Dynamic Contrast-Enhanced Magnetic Resonance Imaging Parameters and Extracellular Volume Fraction as Predictors of Lung Cancer Subtypes and Lymph Node Status in Non-Small-Cell Lung Cancer Patients

**DOI:** 10.7150/jca.88367

**Published:** 2023-09-25

**Authors:** Wenxiu Guo, Binglin Lv, Tao Yang, Mimi Tian, MengXiao Liu, XiangTao Lin, Peng Zhao

**Affiliations:** 1 Department of Radiology, Shandong Provincial Hospital Affiliated to Shandong First Medical University, Jinan, Shandong Province, 250021, China.; 2 Department of Radiology, QiLu Hospital of Shandong University, Jinan, Shandong Province, 250012, China.; 3 Department of Radiology, Shandong Provincial Hospital, Shandong University, Jinan, Shandong Province, 250021, China.; 4 MR Scientific Marketing, Diagnostic Imaging, Siemens Healthineers Ltd., Shanghai, 200126, China.

**Keywords:** Lung cancer, Dynamic contrast-enhanced MRI, Extracellular volume fraction, Lymph node

## Abstract

**Objective:** The aim of this study is to determine whether dynamic contrast-enhanced magnetic resonance imaging (DCE-MRI)-based quantitative parameters and the extracellular volume fraction (ECV) can differentiate small-cell lung cancer (SCLC) from non-small-cell lung cancer (NSCLC), squamous-cell carcinoma (SCC) from adenocarcinoma (Adeno-Ca), and NSCLC with lymph node metastasis from NSCLC without lymph node metastasis.

**Materials and methods:** We prospectively enrolled patients with lung cancer (41 Adeno-Ca, 29 SCC, and 23 SCLC) who underwent DCE-MRI and enhanced T1 mapping prior to histopathological confirmation. Quantitative parameters based on DCE-MRI and ECV based on T1 mapping were compared between SCLC and NSCLC patients, between SCC and Adeno-Ca patients, and between NSCLC patients with and without lymph node metastasis. The area under the receiver-operating characteristic curve (AUC) was used to evaluate the diagnostic performance of each parameter. Spearman rank correlation was used to clarify the associations between ECV and DCE-MRI-derived parameters.

**Results:** Ktrans, Kep, Ve, and ECV all performed well in differentiating SCLC from NSCLC (AUC > 0.729). Ktrans showed the best performance in differentiating SCC from Adeno-Ca (AUC = 0.836). ECV could differentiate NSCLCs with and without lymph node metastases (AUC = 0.764). ECV showed a significant positive correlation with both Ktrans and Ve.

**Conclusions:** Ktrans is the most promising imaging parameter to differentiate SCLC from NSCLC, and Adeno-Ca from SCC. ECV was helpful in detecting lymph node metastasis in NSCLC. These imaging parameters may help guide the selection of lung cancer treatment.

## Introduction

Lung cancer is one of the most common types of cancer, and is further divided into small-cell lung cancer (SCLC) and non-small cell lung cancer (NSCLC). NSCLCs account for approximately 85% of all lung cancers, and are primarily divided into squamous cell carcinoma (SCC) and adenocarcinoma (Adeno-Ca) 1. SCLC differs greatly from NSCLC in terms of biological behavior, treatment, and prognosis. SCLC is more aggressive with a poorer prognosis, and its treatment mainly consists of radiotherapy and chemotherapy, while the treatment of NSCLC includes surgical resection or a combination of radiotherapy and chemotherapy 2. Adeno-Ca and SCC also differ greatly in terms of the choice of chemotherapy and targeted drugs 3. Therefore, the accurate identification of lung cancer subtypes is important for selecting the appropriate treatment. In addition, the accurate preoperative prediction of lymph node metastasis in patients with NSCLC is essential for making treatment-related decisions and assessing patient prognosis. Lymph node metastasis is the most common form of metastasis in NSCLC, and is an important factor affecting staging 4. The preoperative evaluation of lymph node metastases can assist clinicians in determining treatment options and the scope of surgery for NSCLC patients.

Biopsy is the most accurate method to distinguish the pathological subtypes of malignant cancers and to determine the lymph node status; however, biopsy is an invasive examination, and cannot assess the lesion as a whole 5. Conventional computed tomography (CT) and magnetic resonance imaging (MRI) provide only basic information about the lesion, which is insufficient to distinguish the pathological subtypes and predict the lymph node status. Therefore, there is a need to find more useful imaging biomarkers for lung cancer to help clinical decision-making in a comprehensive manner.

Studies have demonstrated that different pathological subtypes of lung cancer show different characteristics with regard to angiogenesis 6, and that lymph node metastasis is associated with the tumor microenvironment 7. Recently, dynamic contrast-enhanced (DCE)-MRI parameters and the extracellular volume fraction (ECV) derived from enhanced T1 mapping have received significant attention as potential tools to reflect the tumor microenvironment 8, 9. Several studies have demonstrated that DCE-MRI can evaluate microvascular density and quantitatively reflect tumor angiogenesis 10. In particular, the DCE-MRI parameter Ktrans has been identified as a reliable marker that reflects cellularity and angiogenesis in various tumors 8. Studies have shown that quantitative DCE-MRI parameters perform well in differentiating malignant lung cancer from benign disease 8 and predicting lymph node status in patients with breast cancer 11. The ECV represents the extracellular interstitial volume as a percentage of the overall tissue volume 9, and mainly reflects the size of the extracellular space. ECV can identify lymphovascular interstitial invasion in cervical cancer patients 12. Therefore, we hypothesized that the pathological subtype of lung cancer and the lymph node status of NSCLC patients could be distinguished using quantitative DCE-MRI parameters and ECV. However, to date, few studies have focused on the value of DCE-MRI parameters in identifying different types of lung cancers, and the role of ECV in lung cancer has not yet been evaluated.

Therefore, the aim of this study was to determine the roles of ECV and DCE-MRI parameters in the identification of lung cancer subtypes and the evaluation of lymph node metastasis in NSCLC patients in order to help clinicians select appropriate treatment strategies.

We have presented this article in accordance with the STARD reporting checklist.

## Materials and Methods

### Patients

**Figure 1:**
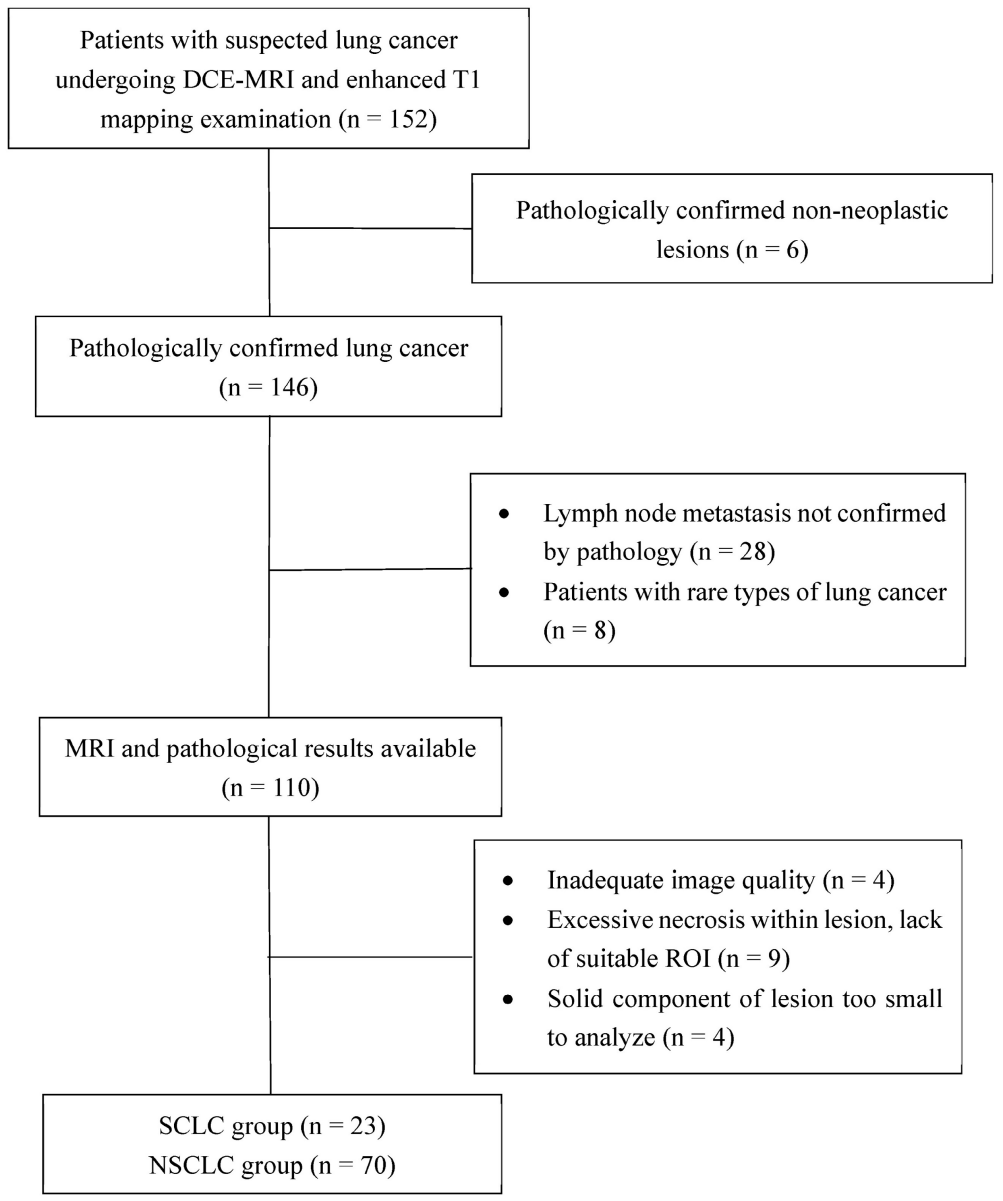
Flowchart of patient selection. DCE-MRI, dynamic contrast enhanced-magnetic resonance imaging; ROI, region of interest; SCLC, small-cell lung cancer; NSCLC, non-small-cell lung cancer

This study was approved by the local institutional review board, and performed in accordance with the ethical standards of the Declaration of Helsinki. Between September 2020 and December 2022, 152 patients with suspected lung cancer were consecutively enrolled in this prospective trial. Informed consent was obtained from all patients. Each patient underwent a series of MRI examinations, which included DCE-MRI and enhanced T1 mapping sequences. The inclusion criteria were as follows: (I) patients with suspected lung cancer on radiography or CT, (II) patients with complete baseline information, (III) patients who had not undergone histopathological examination or anti-tumor therapy prior to the MRI examination, and (IV) patients with lesions ≥1.5 cm in diameter. The pathological subtype of the lesion and the presence or absence of lymph node metastasis were determined using pathological findings. The exclusion criteria were as follows: (I) severe distortion of MRI scans and (II) extensive necrosis and internal tumor hemorrhage, leaving no suitable target area to draw a region of interest (ROI) > 50 mm^2^. All patients underwent MRI scanning within 1 week before histopathological examination or anti-tumor therapy. A flow chat of patient enrollment is shown in Figure 1.

### MRI techniques

MRI scans were obtained using a 3.0-T clinical magnetic resonance scanner (Siemens Prisma 3.0T) with a dedicated 18-channel body-phased array coil. Prior to the examination, patients underwent respiratory training. The patients were in a supine position and entered the scanner head first. T2-half Fourier single-shot turbo spin-echo (T2-HASTE) coronal sequences and segmental transverse T2 BLADE sequences were performed first for lesion localization. Axial thoracic DCE-MRI was performed using a K‑space radial stack‑of-star acquisition scheme with volumetric interpolated breath-hold examination (star‑VIBE sequence). Before DCE-MRI scanning, axial T1 mapping sequences were obtained using B1 inhomogeneity-corrected variable flip angle 3D T1-weighted VIBE sequences with dual flip angles (3° and 15°) to generate a T1 map, which had the same scan location information and the same field of view (FOV) as the DCE-MRI sequence. The acquisition time of the DCE-MRI sequence was 260 s with a temporal resolution of 10 s. After completing the first measurements, we used a high-pressure syringe to inject a 0.15-mmol/kg dose of Gd-DTPA at a flow rate of 2.5 mL/s and followed by a 20-mL saline flush at the same rate. Uninterrupted image acquisition was performed while the contrast was injected, and a total of 25 sequences were collected after contrast injection. Post-contrast T1-mapping images were taken 10 min after contrast-medium injection. The detailed parameters of the MRI scans are listed in Table 1.

### Imaging analysis

All the acquired data were transferred to the syngo.via workstation (Siemens, VB20A). Two radiologists (Drs. A and B, with 3 and 15 years of experience in the CT and MRI diagnosis of thoracic tumors, respectively), who were unaware of the clinical data and histopathological information, except for the diagnosis of lung cancer, analyzed and measured all the scans independently.

The tissue perfusion analysis software module “Tissue 4D” provided in the syngo.via workstation was used to extract the Ktrans, Kep, and Ve values from the DCE-MRI dataset. A population-based arterial input function was used for Tofts modeling to analyze the pharmacokinetic parameters, and no arterial ROIs were needed. Following motion correction and elastic registration performed by the syngo.via workstation, the ROI was independently delineated by the 2 aforementioned radiologists. The radiologists drew the tumor ROIs first, and then, the software produced voxel-based maps for the ROIs. The ROIs were placed over the lesion in the slice with the maximum tumor cross-sectional area and in the slices superior and inferior to the slice with the maximum tumor area; during ROI placement, care was taken to avoid areas of vascularization and necrosis. Images from each patient's CT and MRI sequences (T2-HASTE and fat-saturated T2-BLADE) were referenced during the process of outlining the ROIs.

The perfusion-related parameters Ktrans, Kep, and Ve, were automatically generated by the software. The mean values of these quantitative parameters within the ROIs were recorded. ROIs with the same location, size, and shape were placed on the postcontrast T1 maps in the same way, and copied onto the same section of the non-contrast T1-mapping images. T1 values before and after contrast agent injection (T1pre and T1post) were measured and recorded. Additional ROIs (size, 15 mm^2^) were placed in the aorta to obtain the T1 values of the artery before and after the injection of the contrast agent (T1_blood pre_ and T1_blood post_). The hematocrit obtained from blood samples taken within 3 days of the MRI scan was recorded. The ECV was calculated as follows:

ECV = (1 - hematocrit) × [(1/T1_post_ - 1/T1_pre_)/(1/T1_bloodpost_ - 1/T1_bloodpre_)]

### Statistical analysis

Data were tested for normality and homoscedasticity using the Shapiro-Wilk test and Levene *t*-test, respectively. All continuous numerical data were expressed as the mean ± standard deviation. Between-group differences in parameters were analyzed using the Student *t* test or Mann-Whitney *U*-test. The associations of DCE-MRI parameters and ECV with different tumor subtypes and lymph node status in NSCLC were indicated using box-and-whisker plots. Receiver operating characteristic (ROC) curve analyses were performed for parameters with statistically significant between-group differences to determine the optimal cut-off values of these parameters for predicting SCLC, SCC, and lymph node metastases. The area under the ROC curve (AUC), sensitivity, specificity, and Youden index were calculated. The DeLong test was used to compare the AUCs of different parameters. The interclass correlation coefficient (ICC) was used to assess the reproducibility of the readers' parameter measurements. The average value from the ROIs drawn by each radiologist on 3 slices for each tumor was taken as that physician's measurement for the ICC analysis. The mean value of the 2 radiologists' measurements was used as the final quantitative result for the ROC curve analyses. The Spearman correlation test was applied to explore the correlations between parameters.

## Results

### Baseline information of the patients

**Figure 2:**
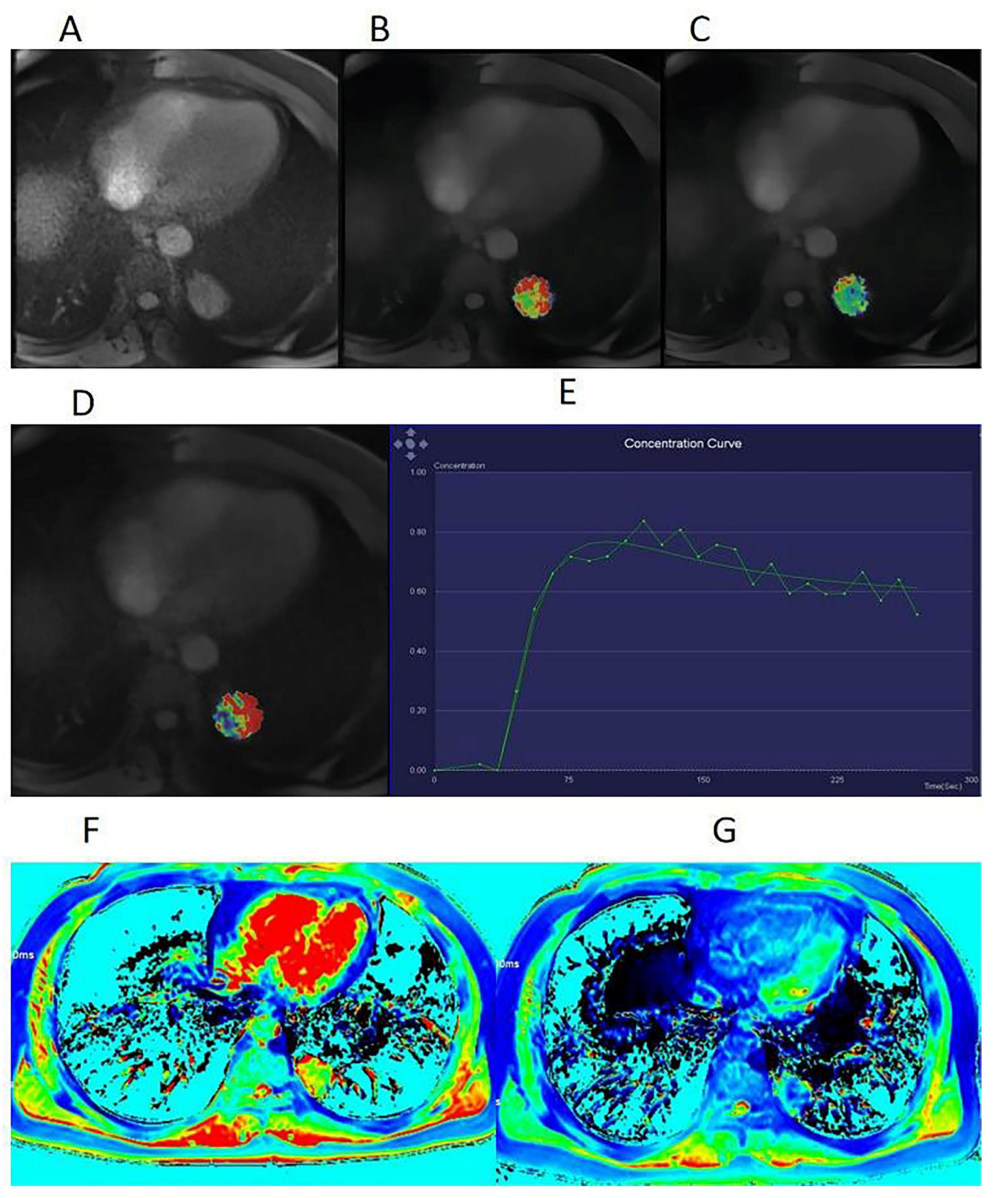
A 53-year-old man with squamous cell carcinoma (SCC). **(A)** Dynamic contrast-enhanced magnetic resonance imaging (DCE-MRI) scan before contrast injection, showing a lesion with a diameter of 33 mm in the upper lobe of the left lung. Volume transfer coefficient (Ktrans) map (B), rate constant (Kep) map (C), extracellular extravascular volume fraction (Ve) map (D), and mean DCE time course curve (E) obtained from the quantification of DCE-MRI scans. The tumor extracellular volume (ECV) fraction was 0.32, as calculated using region-of-interest measurement within the tumor and the aorta on a pseudo-color native T1 map (F) and a pseudo-color enhanced T1 map (G).

A total of 93 patients, including 74 men and 19 women, were enrolled in this study. The age of the patients ranged from 45 years to 76 years, with a mean age of 61.61 ± 6.88 years. The mean initial size of the primary tumor was 3.64 ± 1.66 cm (range, 1.50-7.75 cm). Table 2 summarizes the characteristics of the enrolled patients. All 93 patients were diagnosed with malignant lung cancers on histopathology, including 23 patients with SCLC and 70 patients with NSCLC (29 with SCC and 41 with Adeno-Ca; 28 with lymph node metastasis and 42 without lymph node metastasis). For each patient, the final diagnosis was confirmed by the histopathological examination of either a surgical or biopsy specimen. All SCC patients in this study were men, which is correlated with the fact that the incidence of SCC is much higher in men than in women 13-14. Typical cases are displayed in Figures 2, S1, and S2.

### Interobserver variability

The interobserver agreement ranged from good to excellent for Ktrans (ICC, 0.893; 95% confidence interval [CI]: 0.838-0.929), Kep (ICC, 0.894; 95% CI: 0.840-0.930), Ve (ICC, 0.902; 95% CI: 0.852-0.935), and ECV (ICC, 0.863; 95% CI: 0.793-0.909).

### Roles of DCE-MRI and ECV in differentiating SCLC from NSCLC

**Figure 3:**
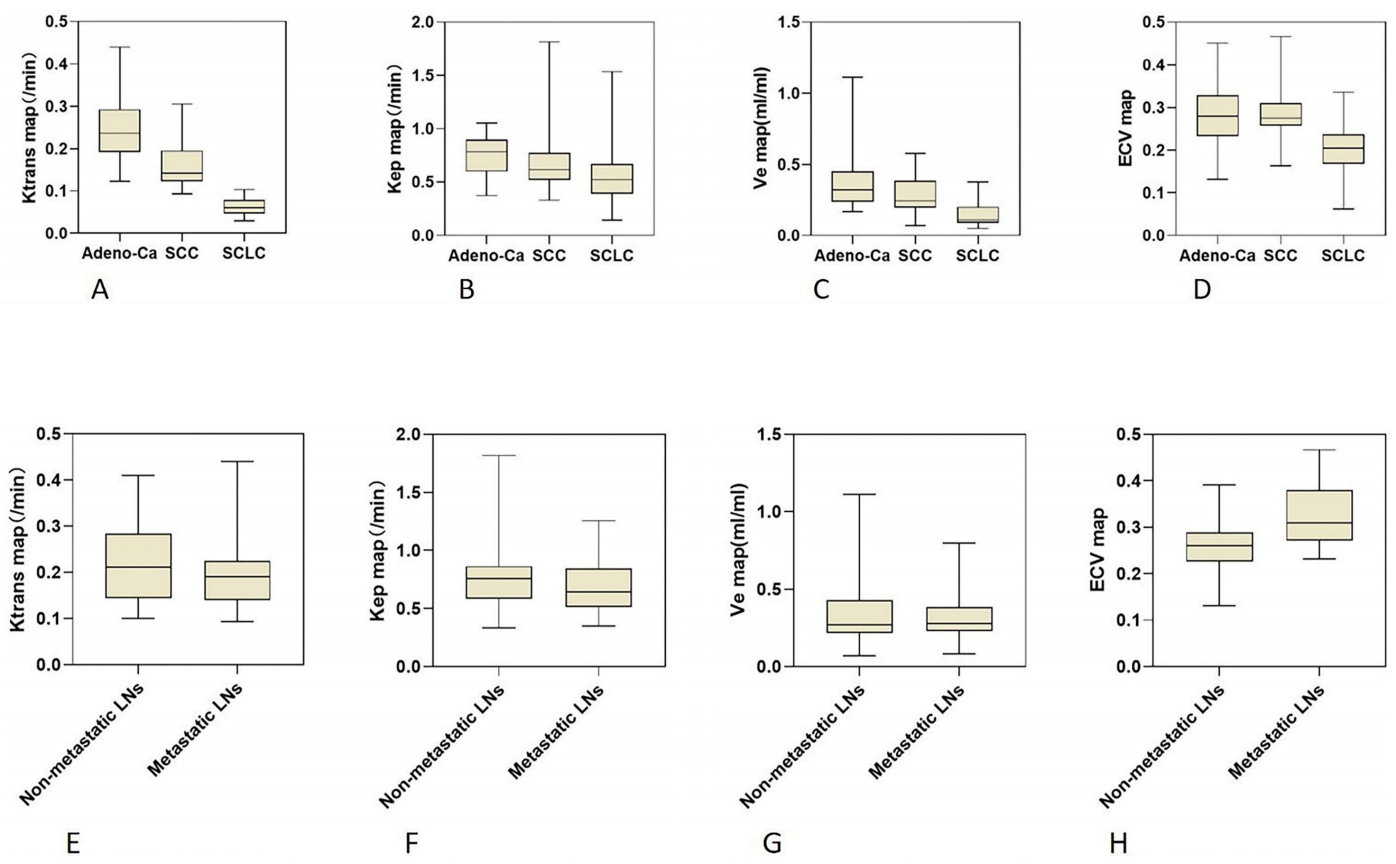
Box plots showing the distribution of tumor DCE-MRI parameter values **(A-C, E-G)** and ECV fractions **(D, H)** among patients with Adeno-Ca, SCC, and SCLC **(A-D)**, and NSCLC patients with and without lymph node metastasis **(E-H)**. DCE-MRI, dynamic contrast-enhanced magnetic resonance imaging; ECV, extracellular volume fraction; Adeno-Ca, adenocarcinoma; SCC, squamous-cell carcinoma; SCLC, small-cell lung cancer; NSCLC, non-small-cell lung cancer; Ktrans, volume transfer coefficient; Kep, rate constant; Ve, extracellular extravascular volume fraction

**Figure 4:**
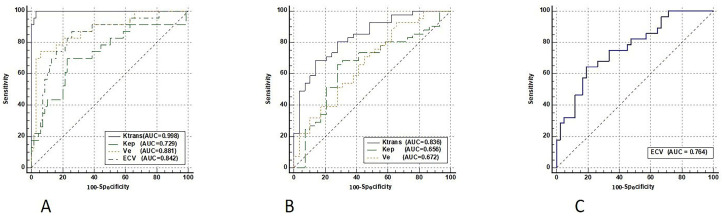
ROC curves of DCE-MRI parameters and ECV fraction for differentiating SCLC from NSCLC **(A)**, Adeno-Ca from SCC **(B)**, and NSCLC with lymph node metastasis from NSCLC without lymph node metastasis **(C)**. ROC, receiver operating characteristic; DCE-MRI, dynamic contrast-enhanced magnetic resonance imaging; ECV, extracellular volume fraction; Adeno-Ca, adenocarcinoma; SCC, squamous-cell carcinoma; SCLC, small-cell lung cancer; NSCLC, non-small-cell lung cancer; Ktrans, volume transfer coefficient; Kep, rate constant; Ve, extracellular extravascular volume fraction; AUC, area under the ROC curve

The DCE-MRI parameters (Ktrans, Kep, and Ve) and ECV characteristics of 3 different histological tumor subtypes were displayed in box plots (Figure 3). Both the DCE-MRI parameters (Ktrans: 0.065 ± 0.020 *vs*. 0.214 ± 0.086, Kep: 0.582 ± 0.337 *vs*. 0.724 ± 0.233, and Ve: 0.142 ± 0.086 *vs*. 0.331 ± 0.178) and ECV (0.197 ± 0.061 *vs*. 0.285 ± 0.069) were significantly lower in SCLC than in NSCLC (*P* < 0.01 for all, Table 3). Ktrans showed perfect diagnostic accuracy to discriminate between SCLC and NSCLC, with an AUC of 0.998 (Table 4, Figure 4). The Delong test showed that Ktrans was significantly superior to the other quantitative parameters in this study (all *P* < 0.0075, Z: 2.672-4.017), and that there was no difference in diagnostic efficacy between Ve and ECV (*P* = 0.589).

### Roles of DCE-MRI and ECV in differentiating Adeno-Ca from SCC

Ktrans was significantly higher in Adeno-Ca than in SCC (0.252 ± 0.087 *vs*. 0.160 ± 0.049, *P* < 0.01). Kep and Ve were also significantly higher in Adeno-Ca than in SCC. ECV did not significantly differ between Adeno-Ca and SCC (Table 3). Ktrans showed the highest AUC value of 0.836 (Table 4), which was significantly higher than that of Kep (DeLong test: Z = 2.076, *P* = 0.038) and Ve (DeLong test: Z = 3.632, *P* < 0.001).

### Roles of DCE-MRI and ECV in differentiating NSCLC with and without lymph node metastasis

Only ECV was able to differentiate NSCLCs with different lymph node statuses (Table 3, Figure 3). The ECV was significantly higher for NSCLC with lymph node metastasis than for NSCLC without lymph node metastasis (0.325 ± 0.068 *vs*. 0.259 ± 0.056, *P* < 0.001). With a cut-off value of 0.293, ECV showed a sensitivity of 64.3% and a specificity of 81.0% to discriminate NSCLC with lymph node metastasis from NSCLC without lymph node metastasis (AUC: 0.764, Table 4, Figure 4).

### Association between ECV and DCE-MRI parameters

The ECV showed a significant positive correlation with Ktrans (Spearman coefficient = 0.329, *P* = 0.001) and Ve (Spearman coefficient = 0.318, *P* = 0.002).

## Discussion

In the current study, we performed both DCE-MRI and enhanced T1 mapping scans for each patient, and investigated the roles of Ktrans, Kep, Ve, and ECV in differentiating between SCLC and NSCLC, between Adeno-Ca and SCC, and between NSCLC with and without lymph node metastasis. Our results showed that DCE-MRI parameters could effectively identify the histopathological subtypes of lung cancer, especially the parameter Ktrans. ECV also played a satisfactory role in the identification of SCLC and NSCLC. Finally, our study provided preliminary evidence of the role of ECV in differentiating NSCLC with and without lymph node metastasis.

The accurate differentiation of the pathological subtypes of lung cancer is critical for selecting treatment options. Our study showed that the DCE-MRI-based Ktrans, Kep, and Ve values and the T1-mapping-based ECV were significantly lower in SCLC than in NSCLC. This could be explained by the histopathological and cytological characteristics of SCLC. SCLC has a higher vascularization in its early stages 15, which is consistent with the early development of widespread metastatic disease in patients with SCLC.

In contrast, the advanced stages of SCLC are associated with markedly less angiogenesis, and respond poorly to chemotherapy-associated antiangiogenic drugs 16. The SCLC patients in our study all had relatively advanced-stage disease, which is consistent with the report that SCLC is frequently clinically advanced at the time of diagnosis 16. Thus, the Ktrans and Kep parameters, which indicate immature neovascularization, were low in the SCLC patients. One of the most common cytological features of SCLC is that the tumor usually consists of small, round or ovoid cancer cells with high cell density 17. Both Ve and ECV may reflect the degree of cellularity of the tissue within the ROI 18. As the degree of cellularity increases, the extracellular space decreases, and the Ve and ECV also decrease. This explains why Ve and ECV were extremely low in SCLC in this study.

Our study also showed that Ktrans was significantly higher in Adeno-Ca than in SCC, which is consistent with the findings of Zhang et al. 19 This may be a result of the difference in microvascular density between these 2 lung cancer subtypes. Vascular endothelial growth factor (VEGF) expression has been shown to be significantly higher in Adeno-Ca cells than in SCC cells, and the number of microvessels is higher in Adeno-Ca than in SCC, implying a higher capacity for angiogenesis and higher vascular permeability in Adeno-Ca than in SCC.

The accurate preoperative evaluation of lymph node status is important for determining patient prognosis, improving survival rates, and improving quality of life 4. Our study found that patients with lymph node metastases had a higher ECV than those without metastases. This is consistent with the study by Wang et al. 12, which found a significantly higher ECV in patients with invasion of the lymphovascular space than in those without lymphovascular invasion. The low resistance of tumor tissues to transcapillary fluid flow leads to the fluid in the capillaries entering and accumulating in the interstitium 20, 21, resulting in a larger extracellular space, and increased tissue rigidity and cellular hydraulic pressure, which facilitate the entry of tumor cells into small blood vessels and lymphatic vessels 22. The larger the extracellular space, the greater the interstitial hydraulic pressure, and the greater the likelihood of tumor cell metastasis through small vessels and lymphatic vessels. We consider that this may account for the higher ECV in NSCLCs with lymph node metastases than in NSCLCs without lymph node metastases.

Tumor cells directly invade the microvasculature, enter the tumor mesenchyme through the incomplete basement membrane, and then enter the lymphatic vessels, causing lymph node metastasis 7*.* Based on the above theory, parameters reflecting microvascular features might predict lymph node metastasis. Gao et al. reported that DCE-MRI parameters were potential predictors of axillary lymph node metastasis in breast cancer 11. However, we did not observe significant differences in DCE-MRI parameters between lung cancer patients with and without lymph node metastasis. This discrepancy may be due to the following reasons: (1) There exist differences in biological behavior between breast cancer and lung cancer 23. (2) DCE-MRI scans of the lungs are more challenging, and are affected by more factors, such as respiratory and cardiac artifacts. (3) Finally, the scanning techniques and parameters were not uniform between the 2 studies. However, few studies have investigated the roles of quantitative DCE-MRI parameters in predicting lymph node metastasis in lung cancer, and studies with expanded sample sizes are needed to validate our findings.

In our study, Ve was found to correlate with ECV, but the correlation was not as good as expected. This may be due to the following reasons: (1) Ve and ECV are not exactly the same. ECV differs from Ve in that it also includes the intravascular space. Lung cancer has a complex blood supply, and the fraction of intravascular space in lung tumors may be highly variable between individuals 24. (2) The 2 parameters are based on different mechanisms. (3) Finally, Ve obtained from DCE-MRI is influenced by pharmacokinetic models.

The limitations of this study should be acknowledged. First, this was a single-center study with a small sample size; we will further expand the sample size, and validate the results in a multi-center setting. Second, there is no uniform standard for the optimal ECV acquisition time for lung cancer, and the trigger time in this study was based on data from early liver experiments 25. Studies 25-26 have shown that a dynamic equilibrium is usually established within 10 min of contrast-agent administration, after which the accuracy of ECV measurements is constant for a certain period of time. Therefore, in this study, the post-contrast T1 mapping images were taken at 10 min after contrast-medium injection in all patients. Third, we used the mean value of the ROI to define the measured parameters, but a histogram analysis of these heterogeneous lesions may have been more useful to identify differences between lesions.

## Conclusion

Quantitative DCE-MRI parameters as well as ECV are potential imaging biomarkers for differentiating the pathological subtypes of lung cancer. Moreover, ECV can be used to predict the lymph node metastasis status in NSCLC patients. These parameters can guide the treatment of lung cancer patients.

## Supplementary Material

Figures S1 and S2.

## Figures and Tables

**Table 1:** MRI parameters

Sequence	Repetition time (ms)	Echo time (ms)	Section thickness (mm)	Layer spacing (mm)	Field of view (mm^2^)	Scanning time (s)	Respiratory compensation
T2-HASTE	1200	96	3.5	1.0	400 × 400	36	Hold breath
Fat-saturated T2-BLADE	2890	94	4.0	0.8	380 × 380	68	Hold breath
Native T1 mapping	5.01	2.3	2.5	0.8	380 × 265	14	Hold breath
DCE-MRI	2.51	1.13	3.0	0.6	220 × 220	300	Breathe freely
Enhanced T1 mapping	5.01	2.3	2.5	0.8	380 × 265	14	Hold breath

T2-HASTE, half Fourier single-shot turbo spin-echo; DCE-MRI, dynamic contrast-enhanced magnetic resonance imaging

**Table 2:** Clinicopathological characteristics of patients included in this study

	Adeno-Ca (N = 41)	SCC (N = 29)	SCLC (N = 23)
Age at diagnosis (yrs), mean ± SD	61.15 ± 7.00	60.14 ± 6.62	64.30 ± 6.18
Sex			
Male	25	29	20
Female	16	0	3
Lymph node metastasis			
Present	15	13	15
Absent	26	16	8
Lesion location			
RUL	13	9	11
RML	2	3	3
RLL	9	0	2
LUL	8	13	6
LLL	9	4	1
Lesion size (cm), mean ± SD	2.94 ± 1.31	4.24 ± 1.70	4.13 ± 1.72

Adeno-Ca, adenocarcinoma; SCC, squamous-cell carcinoma; SCLC, small-cell lung cancer; SD, standard deviation; RUL, right upper lobe; RML, right middle lobe; RLL, right lower lobe; LUL, left upper lobe; LLL, left lower lobe

**Table 3:** Comparison of quantitative DCE-MRI parameters and ECV in different pathological subtypes of lung cancer and in NSCLC patients with and without lymph node metastasis

Parameter	SCLC (n = 23)	NSCLC (n = 70)	NSCLC			
			Adeno-Ca (n = 41)	SCC (n = 29)	No metastasis (n = 42)	Metastasis (n = 28)
Ktrans	0.065 ± 0.020^a^	0.214 ± 0.086	0.252 ± 0.087^b^	0.160 ± 0.049	0.227 ± 0.089	0.195 ± 0.078
Kep	0.582 ± 0.337^a^	0.724 ± 0.233	0.752 ± 0188^b^	0.686 ± 0.285	0.751 ± 0.247	0.684 ± 0.209
Ve	0.142 ± 0.086^a^	0.331 ± 0.178	0.372 ± 0.199^b^	0.274 ± 0.124	0.341 ± 0.198	0.316 ± 0.145
ECV	0.197 ± 0.061^a^	0.285 ± 0.069	0.284 ± 0.072	0.286 ± 0.065	0.259 ± 0.056	0.325 ± 0.068^c^

^a^*P* < 0.003 between SCLC and NSCLC, with the Student *t*-test or Mann-Whitney *U*-test. ^b^*P* < 0.003 between SCC and Adeno-Ca, with the Mann-Whitney *U*-test. ^c^*P* < 0.001 between NSCLC patients with and without lymph node metastasis, with the independent-samples *t*-test. DCE-MRI, dynamic contrast-enhanced magnetic resonance imaging; ECV, extracellular volume fraction; SCLC, small-cell lung cancer; NSCLC, non-small cell lung cancer; Adeno-Ca, adenocarcinoma; SCC, squamous-cell carcinoma

**Table 4:** Effectiveness of multi-parametric MRI for differentiating between pathological subtypes of lung cancer and between patients with and without lymph node metastasis

Parameter	AUC	Cut-off	Sensitivity (%)	Specificity (%)	Youden index
SCLC *vs*. NSCLC					
Ktrans	0.998 (0.994-1)	0.105	97.1	1	0.971
Kep	0.729 (0.600-0.858)	0.551	77.1	69.6	0.467
Ve	0.881 (0.795-0.967)	0.146	94.3	73.9	0.682
ECV	0.842 (0.747-0.937)	0.246	74.3	87.0	0.613
Adeno-Ca *vs*. SCC					
Ktrans	0.836 (0.743-0.929)	0.208	68.3	86.2	0.545
Kep	0.656 (0.523-0.789)	0.737	65.9	72.4	0.383
Ve	0.672 (0.543-0.801)	0.217	90.2	37.9	0.281
NSCLC with *vs*. without lymph node metastasis					
ECV	0.764 (0.652-0.877)	0.293	64.3	81.0	0.453

MRI, magnetic resonance imaging; AUC, area under the receiver operating characteristic curve; SCLC, small-cell lung cancer; NSCLC, non-small cell lung cancer; Adeno-Ca, adenocarcinoma; SCC, squamous-cell carcinoma; ECV, extracellular volume fraction

## Abbreviations

Adeno-Ca: adenocarcinoma
AUC: area under the receiver operating characteristic curve
DCE-MRI: dynamic contrast-enhanced magnetic resonance imaging
ECV: extracellular volume fraction
FOV: field of view
ICC: interclass correlation coefficient
NSCLC: non-small-cell lung cancer
ROC: receiver operating characteristic
ROI: region of interest
SCLC: small-cell lung cancer
SCC: squamous-cell carcinoma
T2-HASTE: T2-half Fourier single-shot turbo spin-echo
VEGF: vascular endothelial growth factor
