# Supplementary material for: Role of Dynamic Contrast-Enhanced Magnetic Resonance Imaging Parameters and Extracellular Volume Fraction as Predictors of Lung Cancer Subtypes and Lymph Node Status in Non-Small-Cell Lung Cancer Patients
Source: J Cancer. 2023 Sep 25;14(16):3108–16. doi: 10.7150/jca.88367 (PMC10583593; doi:10.7150/jca.88367)
Supplement: Supplementary file 1 — Figures S1 and S2. [file jcav14p3108s1.pdf]

Supplementary material

Figures S1 and S2

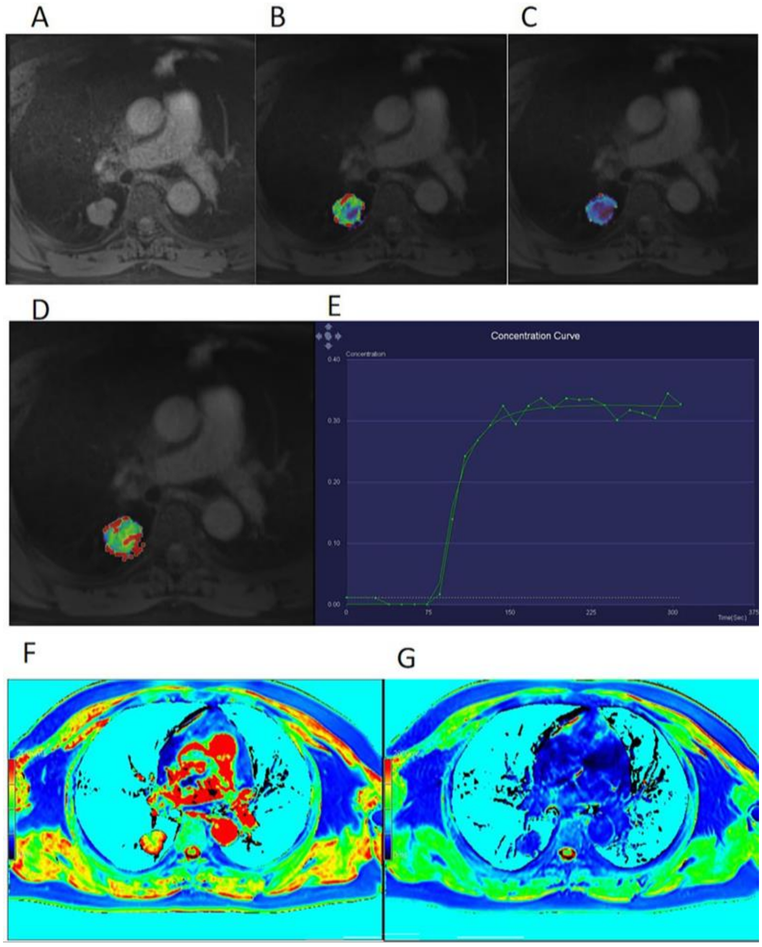

Figure S1 A 64-year-old man with adenocarcinoma (Adeno-Ca). (A) Dynamic contrast-enhanced magnetic resonance imaging (DCE-MRI) scan before contrast injection, showing a lesion with a diameter of 19 mm in the lower lobe of the right lung.

Volume transfer coefficient (Ktrans) map (B), rate constant (Kep) map (C), extracellular extravascular volume fraction (Ve) map (D), and mean DCE time course curve (E) obtained from the quantification of DCE-MRI scans. The tumor extracellular volume (ECV) fraction was 0.35, as calculated using region-of-interest measurement within the tumor and the aorta on a pseudo-color native T1 map (F) and pseudo-color enhanced T1 map (G).

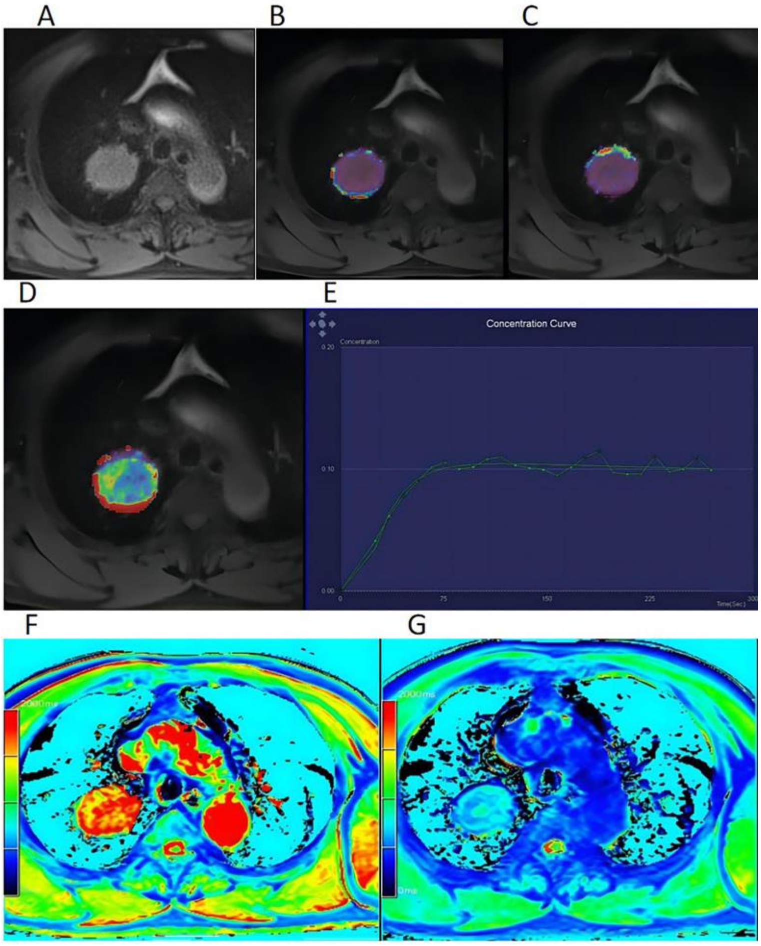

Figure S2 A 64-year-old man with small-cell lung cancer (SCLC). (A) Dynamic contrast-enhanced magnetic resonance imaging (DCE-MRI) scan before contrast injection, showing a lesion with a diameter of 38 mm in the upper lobe of the right lung.

Volume transfer coefficient (Ktrans) map (B), rate constant (Kep) map (C), extracellular extravascular volume fraction (Ve) map (D), and mean DCE time course curve (E) obtained from the quantification of DCE-MRI scans. The tumor extracellular volume (ECV) fraction was 0.20, as calculated using region-of-interest measurement within the tumor and the aorta on a pseudo-color native T1 map (F) and pseudo-color enhanced T1 map (G).
